# Obesity-Related Gut Microbiota Aggravates Alveolar Bone Destruction in Experimental Periodontitis through Elevation of Uric Acid

**DOI:** 10.1128/mBio.00771-21

**Published:** 2021-06-01

**Authors:** Keisuke Sato, Kyoko Yamazaki, Tamotsu Kato, Yumiko Nakanishi, Takahiro Tsuzuno, Mai Yokoji-Takeuchi, Miki Yamada-Hara, Nobuaki Miura, Shujiro Okuda, Hiroshi Ohno, Kazuhisa Yamazaki

**Affiliations:** a Research Unit for Oral-Systemic Connection, Division of Oral Science for Health Promotion, Niigata University Graduate School of Medical and Dental Sciences, Chuo-ku, Niigata, Japan; b Division of Periodontology, Niigata University Graduate School of Medical and Dental Sciences, Chuo-ku, Niigata, Japan; c Laboratory for Intestinal Ecosystem, RIKEN Centre for Integrative Medical Sciences (IMS), Yokohama, Kanagawa, Japan; d Intestinal Microbiota Project, Kanagawa Institute of Industrial Science and Technology, Kawasaki, Japan; e Division of Bioinformatics, Niigata University Graduate School of Medical and Dental Sciences, Chuo-ku, Niigata, Japan; Brigham and Women's Hospital/Harvard Medical School

**Keywords:** gut microbiome, metabolomics, obesity, periodontal disease, uric acid

## Abstract

Obesity is a risk factor for periodontal disease (PD). Initiation and progression of PD are modulated by complex interactions between oral dysbiosis and host responses. Although obesity is associated with increased susceptibility to bacterial infection, the detailed mechanisms that connect obesity and susceptibility to PD remain elusive. Using fecal microbiota transplantation and a ligature-induced PD model, we demonstrated that gut dysbiosis-associated metabolites from high-fat diet (HFD)-fed mice worsen alveolar bone destruction. Fecal metabolomics revealed elevated purine degradation pathway activity in HFD-fed mice, and recipient mice had elevated levels of serum uric acid upon PD induction. Furthermore, PD induction caused more severe bone destruction in hyperuricemic than normouricemic mice, and the worsened bone destruction was completely abrogated by allopurinol, a xanthine oxidase inhibitor. Thus, obesity increases the risk of PD by increasing production of uric acid mediated by gut dysbiosis.

## INTRODUCTION

Obesity is a major cause of many health complications and a risk factor for periodontal disease (PD). Obese individuals have increased odds of 1.1 to 4.5 for the presence of inflammation or periodontal destruction (1). PD is characterized by connective tissue attachment destruction and alveolar bone resorption. It is initiated by dysbiosis of oral bacteria, particularly periodontopathic bacteria such as Porphyromonas gingivalis (2). Disease progression involves complex interactions between bacteria and host responses. However, the detailed mechanisms that connect obesity and susceptibility to PD are unclear.

Gut dysbiosis is associated with alterations of bacterial metabolites, the host metabolic profile, gut barrier functions, and the gut immune profile, which increase the risk of inflammatory and metabolic diseases. The gut microbiome is influenced by genetics, diet, antibiotics, and lifestyle (3). Among these factors, obesity is intimately and bidirectionally associated with alteration of the gut microbiome. Thus, increased development and severity of PD in obese individuals might be attributable to gut dysbiosis and related phenomena. Several studies have shown the effects of the gut microbiota on the severity of experimental PD in rats. Long-term supplementation with omega-3 fatty acids, which have beneficial effects on gut microbiota (4), in a P. gingivalis-induced experimental PD model reduces alveolar bone loss compared with that of control rats fed corn oil (5).

Probiotic therapy with Bacillus subtilis reduces periodontal tissue destruction in ligature-induced PD concomitant with morphological improvement of the small intestine (6). Furthermore, administration of metformin in drinking water decreases alveolar bone loss in ligature-induced PD. The beneficial effects of metformin are mediated at least in part by its action on the gut microbiota (7). These reports suggest that manipulation of the gut microbiota by probiotics or dietary supplements suppresses tissue destruction in experimental PD models. Additionally, recent evidence has suggested an intramucosal connection between the mouth and gut (8).

Obesity-related pathology is attributable to gut dysbiosis. Thus, the relationship between obesity and PD might be explained by gut dysbiosis and subsequent pathological changes that affect host responses. We clarified whether gut dysbiosis deteriorates tissue destruction and elucidated the underlying mechanisms using fecal microbial transplantation (FMT) from obese and lean mice and a ligature-induced PD model.

## RESULTS

### Obesity-related microbiota worsens bone destruction in experimental PD.

After 1 week of antibiotic treatment, C57BL/6N mice received FMT from either lean or obese mice (fed regular chow [RC] or a high-fat diet [HFD], respectively) and were subjected to ligature-induced experimental PD (Fig. S1A and B). Following FMT, there were no significant differences in the body weight change of recipient mice among the experimental groups (Fig. S1C).

10.1128/mBio.00771-21.1 FIG S1 Experimental design and body weight changes of fecal microbial transplantation (FMT) donor mice, recipient mice, and recipient mice at 1 week after ligature placement/nonplacement. (A) Male C57BL/6 mice were fed either regular chow (RC) or a high-fat diet (HFD) for 8 weeks. Feces collected from these donor mice (referred to as RC and HFD donors, respectively) were transplanted into recipient mice pretreated with antibiotics (referred to as RC-FMT and HFD-FMT, respectively). Control mice received PBS administration instead of feces. FMT-treated mice with or without ligature-induced periodontitis (referred to as RC-FMT without periodontitis [PD], RC-FMT with PD, HFD-FMT without PD, and HFD-FMT with PD) were used in the experiment. The names of the experimental groups are shown in the hatched boxes. (B) A significant difference was observed between RC and HFC donor mice. (C) No difference was observed in recipient mice among the experimental groups. *P* < 0.01; Mann-Whitney *U*-test. Download FIG S1, TIF file, 1.9 MB. Copyright © 2021 Sato et al. 2021 Sato et al. https://creativecommons.org/licenses/by/4.0/ This content is distributed under the terms of the Creative Commons Attribution 4.0 International license.

**FIG 1:**
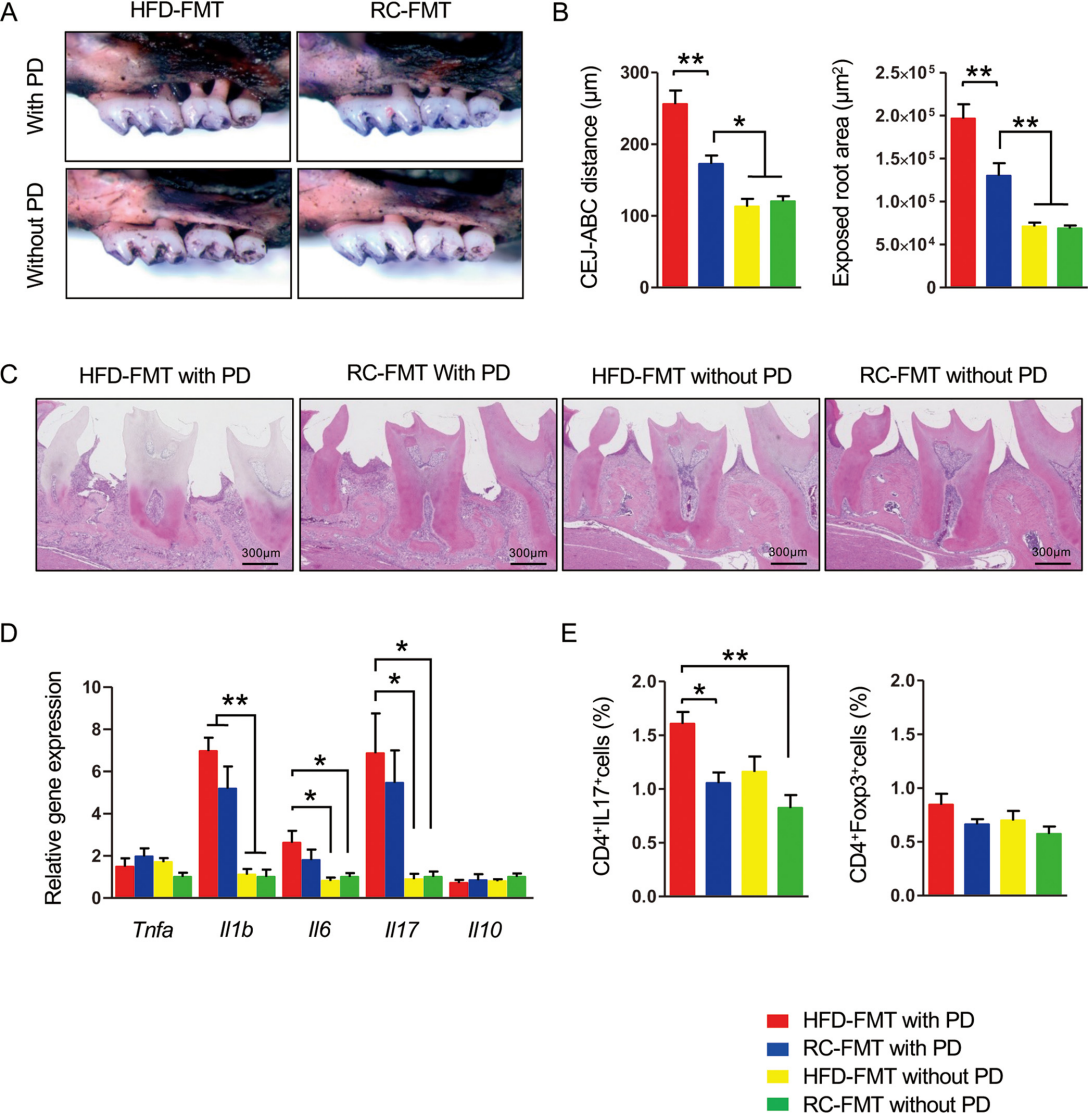
Fecal microbial transplantation (FMT) affects the response of periodontal tissue in ligature-induced periodontitis (PD). Male C57BL/6N mice received FMT from regular chow (RC)- or high-fat diet (HFD)-fed mice and were either subjected to or not subjected to experimental periodontitis by ligature placement on the maxillary second molar (*n* = 5/group). (A) Effects of ligature placement on alveolar bone resorption in mice that received FMT from RC-fed (RC-FMT) or HFD-fed (HFD-FMT) mice. Representative photographs obtained after soft tissue removal are shown. (B) The distance between the cementoenamel junction and alveolar bone crest and the exposed tooth root area of the mesial root of the maxillary second molar was measured under a stereoscopic microscope. Red, HFD-FMT with PD; blue, RC-FMT with PD; yellow, HFD-FMT without PD; green, RC-FMT without PD. (C) Histological findings of gingival tissues of ligated and untreated mice. Sections were stained with hematoxylin and eosin. Representative photographs are shown. (D) Relative gene expression levels in the gingiva of each experimental group. The relative quantity of mRNA was normalized to that of glyceraldehyde-3-phosphate dehydrogenase mRNA. (E) Lymphocyte fractions were obtained from submandibular lymph nodes. Cells were stimulated with PMA and ionomycin at a concentration of 1 × 10^6^/ml. The cells were stained with anti-CD4 and anti-IL-17 antibodies, and 1 × 10^4^ cells were analyzed by flow cytometry. The percentages of CD4^+^IL-17^+^ and CD4^+^FoxP3^+^ cells were compared. Data are expressed as the mean ± standard error of the mean (SEM). *, *P* < 0.05; **, *P* < 0.01; one-way analysis of variance (ANOVA) with Bonferroni’s correction for multiple comparisons.

Ligature placement induced significant alveolar bone destruction regardless of the source of FMT, namely, RC- or HFD-fed mice. However, bone destruction was significantly worse in mice that received FMT from HFD-fed mice (here referred to as HFD-FMT mice) than mice that received FMT from RC-fed mice (here referred to as RC-FMT mice) (Fig. 1A to C). Because the inflammatory response in gingival tissue is critical for tissue destruction in PD, we analyzed inflammatory cytokine gene expression in gingival tissue. Interleukin-6 (IL-6) and IL-17 gene expression in gingival tissue was significantly higher in mice with ligature-induced PD after receiving HFD-FMT than in mice that received FMT regardless of diet without PD. IL-1β gene expression was significantly higher in mice with PD than in those without PD, irrespective of the source of FMT (Fig. 1D). Expression of tumor necrosis factor-α and IL-10 showed no differences. The proportion of Th17 cells in subgingival lymph nodes was significantly higher in HFD-FMT with PD mice than in mice that received RC-FMT with or without PD, which suggested that HFD-FMT rather than induction of PD was involved in the elevated proportion of Th17 cells in these lymph nodes. No difference in regulatory T cells (Treg cells) was observed (Fig. 1E).

To analyze the effect of FMT itself on inflammation in gingival tissue, alveolar bone resorption and gene expression were compared among mice that received FMT from phosphate-buffered saline (PBS)-administered mice (control), RC-fed mice, or HFD-fed mice. Although there was no difference in bone resorption among the experimental groups (Fig. S2A and B), gene expression of IL-17 and IL-10 was significantly higher in RC-FMT and HFD-FMT mice (both received FMT) than in PBS-administered mice, with no difference in terms of the source of FMT. Expression of other genes showed no differences among experimental groups (Fig. S2C).

10.1128/mBio.00771-21.2 FIG S2 FMT affects the gut immune profile, but not periodontal inflammation. Male C57BL/6N mice that received fecal microbial transplantation (FMT) from RC- or HFD-fed mice were analyzed (*n* = 6/group). (A) Effects of FMT on alveolar bone resorption. Images were obtained after soft tissue removal. Representative images are shown. (B) The distance between the cementoenamel junction and alveolar bone crest and the exposed tooth root surface area of the mesial root of the maxillary second molar was measured under a stereoscopic microscope. (C) Comparison of relative gene expression levels in the gingiva. The relative quantity of mRNAs was normalized to that of glyceraldehyde-3-phosphate dehydrogenase mRNA. (D) Effect of FMT on the proportions of Th17 and Treg cells in mesenteric lymph nodes. Lymphocyte fractions were obtained from mesenteric lymph nodes. Cells were stimulated at a concentration of 1 × 10^6^/ml. The cells were stained with anti-CD4, anti-IL-17, and anti-Foxp3 antibodies. Cells (1 × 10^4^) were analyzed by flow cytometry. Representative plots of one experiment with three mice per group are shown. The percentages of double-positive cells are shown. Data are expressed as the mean ± SEM (*n* = 6). *, *P* < 0.05; **, *P* < 0.01; one-way ANOVA with Bonferroni’s correction for multiple comparisons. Download FIG S2, TIF file, 1.9 MB. Copyright © 2021 Sato et al. 2021 Sato et al. https://creativecommons.org/licenses/by/4.0/ This content is distributed under the terms of the Creative Commons Attribution 4.0 International license.

Mice that received FMT from HFD-fed mice (HFD-FMT) had an increased proportion of Th17, but not Treg, cells in mesenteric lymph nodes compared with those that received FMT from RC-fed mice (RC-FMT) (Fig. S2D). In addition to the effect on the gut immune profile, FMT is considered to have some effect on gingival gene expression. However, a substantial effect on gingival gene expression was exerted by PD.

### FMT and PD induction primarily induce changes in gut and oral microbiotas, respectively.

**FIG 2:**
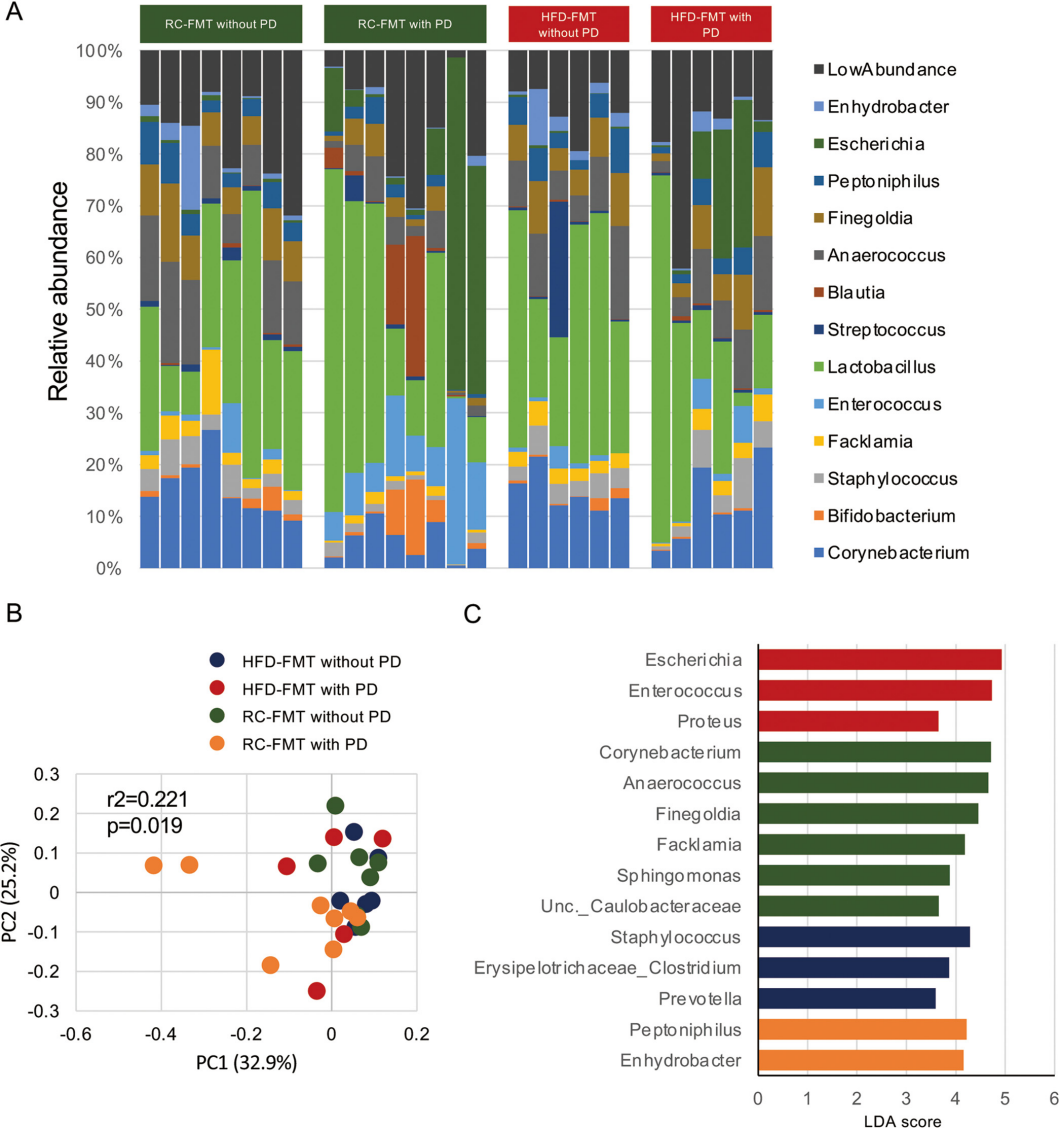
The oral microbiome is affected by ligature-induced periodontitis (PD) and FMT. FMT-treated mice with or without ligature-induced PD (referred to as RC-FMT without PD [*n* = 8], RC-FMT with PD [*n* = 8], HFD-FMT without PD [*n* = 6], and HFD-FMT with PD [*n* = 6]) were used in this experiment. (A) Relative abundance of oral bacterial taxa at the genus level in each experimental group. (B) Principal coordinate analysis (PCoA) score plot of the oral microbiota profiles of the four groups using unweighted UniFrac distance (ANOSIM). (C) Linear discriminant analysis (LDA) scores from LEfSe analysis. Enriched taxa in RC-FMT mice without PD, RC-FMT with PD, HFD-FMT without PD, and HFD-FMT with PD are indicated in green, yellow, blue, and red, respectively.

Next, we examined whether the difference in alveolar bone destruction caused by PD in RC-FMT and HFD-FMT mice was attributable to the difference in the oral microbiota composition (Fig. 2A). As shown in Fig. 2B, the oral microbiota composition was significantly different among experimental groups with or without induction of PD. Linear discriminant analysis effect size (LEfSe) indicated that whereas *Corynebacterium*, *Anaerococcus*, *Facklamia*, *Finegoldia*, *Sphingomonas*, and unclassified *Caulobacteraceae* were more enriched in RC-FMT mice without periodontitis, *Peptoniphilus* and *Enhydrobacter* were enriched in RC-FMT mice with periodontitis. In HFD-FMT mice, Escherichia, *Enterococcus*, and Proteus were enriched in mice with PD, whereas Staphylococcus, *Erysipelotrichaceae*, and *Prevotella* were characteristic taxa in mice without PD (Fig. 2C). Principal-coordinate analysis (PCoA) of unweighted UniFrac distance revealed a significant qualitative difference in oral microbiota composition only between RC-FMT mice with or without induction of PD (Fig. S3A). However, no effect of PD induction was found in HFD-FMT mice (Fig. S3B). Additionally, it became evident that the source of FMT had no effect on the oral microbiota composition (Fig. S3C and D). These results suggested that ligature-induced changes in oral bacterial compositions were not involved in the worsened alveolar bone destruction in HFD-FMT mice.

10.1128/mBio.00771-21.3 FIG S3 Oral microbiome changes after FMT and ligature placement in RC-FMT, but not HFD-FMT, mice. Oral swabs obtained after FMT treatment and again at 1 week after induction of periodontitis were examined. (A) Principal coordinate analysis (PCoA) score plot of the oral microbiota profiles in RC-FMT without (*n* = 8) and with PD (*n* = 8). (B) PCoA score plot of the oral microbiota profiles in HFD-FMT without (*n* = 6) and with PD (*n* = 6) mice. (C) PCoA score plot of oral microbiota in RC-FMT with PD (*n* = 8) and HFD-FMT with PD (*n* = 6) mice. (D) PCoA score plot of oral microbiota in RC-FMT without PD (*n* = 8) and HFD-FMT without PD (*n* = 6) mice. Analyses were conducted using the unweighted UniFrac distance (ANOSIM). Download FIG S3, TIF file, 1.9 MB. Copyright © 2021 Sato et al. 2021 Sato et al. https://creativecommons.org/licenses/by/4.0/ This content is distributed under the terms of the Creative Commons Attribution 4.0 International license.

### Diet and induction of PD modulate gut ecology and metabolites.

**FIG 3:**
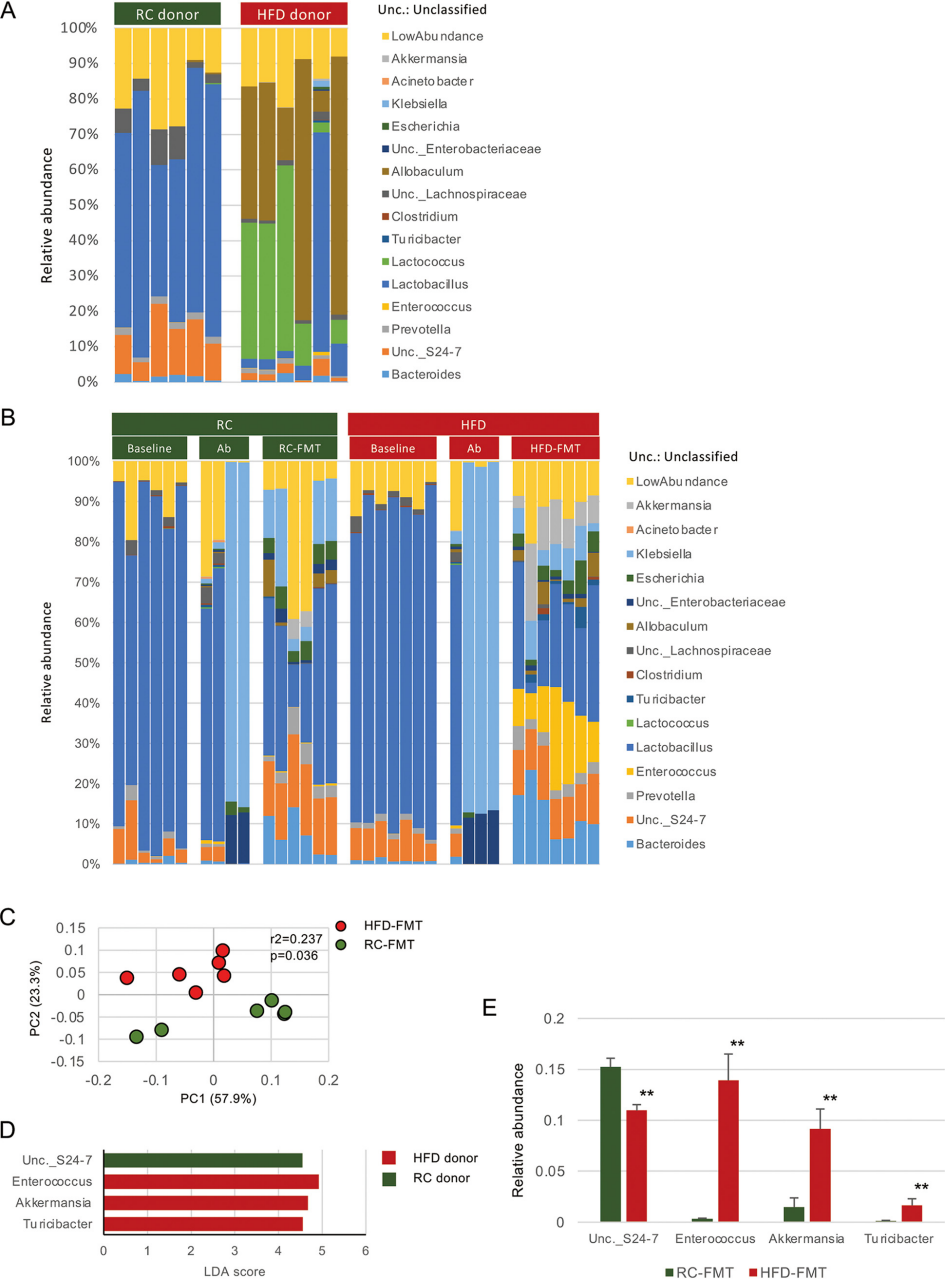
Different diets and FMT from mice fed different diets affect the gut microbiota. Feces obtained before (baseline) and after antibiotic treatment (Ab), after FMT (RC-FMT and HFD-FMT), and from donor mice (RC donor and HFD donor) were used in this experiment. (A) Relative abundance of gut bacterial taxa at the genus level in RC-fed (RC donor) and HFD-fed (HFD donor) mice. (B) Relative abundance of gut bacterial taxa at the genus level at baseline, after antibiotic treatment, and after fecal transplantation from RC-fed and HFD-fed mice. (C) Principal coordinate analysis (PCoA) score plot of the gut microbiota profiles of RC-FMT (*n* = 6) and HFD-FMT (*n* = 6) mice using unweighted UniFrac distance (ANOSIM). FMT from RC-fed and HFD-fed mice significantly affected recipient gut microbiota. (D) LDA scores from LEfSe analysis of fecal gut microbiota RC- and HFD-fed mice. (E) Pairwise comparisons of significantly changed bacterial taxa between RC-FMT and HFD-FMT mice. **, *P* < 0.01; Mann-Whitney *U*-test.

HFD ingestion induced significant changes in the gut microbiota composition compared with RC ingestion (Fig. 3A and Fig. S4A). RC feeding increased the abundance of *Lactobacillus*, unclassified (Unc.) S24-7, Unc. *Erysipelotrichaceae*, *Prevotella*, and *Desulfovibrio*, whereas HFD feeding enriched *Allobaculum*, *Lactococcus*, *Akkermansia*, *Turicibacter*, and *Enterococcus* (Fig. S4B and C).

10.1128/mBio.00771-21.4 FIG S4 The gut microbiome is affected by HFD feeding. Fecal samples from C57BL/6 mice fed either RC (RC donor) or HFD (HFD donor) (*n* = 6 each) were analyzed. (A) Principal coordinate analysis (PCoA) score plot of the gut microbiota profile of RC and HFD donor mice using the unweighted UniFrac distance (ANOSIM). (B) LDA scores from LEfSe analysis. Enriched taxa in RC and HFD donor samples are indicated in green and red, respectively. (C) Pairwise comparisons of significantly changed bacterial taxa between RC and HFD donor mice. *, *P* < 0.05; **, *P* < 0.01; Mann-Whitney *U*-test. Download FIG S4, TIF file, 1.9 MB. Copyright © 2021 Sato et al. 2021 Sato et al. https://creativecommons.org/licenses/by/4.0/ This content is distributed under the terms of the Creative Commons Attribution 4.0 International license.

After FMT, recipient mice demonstrated distinct gut microbiota compositions between RC-FMT and HFD-FMT mice (Fig. 3B and C). The microbiota enriched by each diet appeared to be transmitted from donor to recipient mice, such as Unc. S24-7, *Enterococcus*, *Akkermansia*, and *Turicibacter* (Fig. 3D).

**FIG 4:**
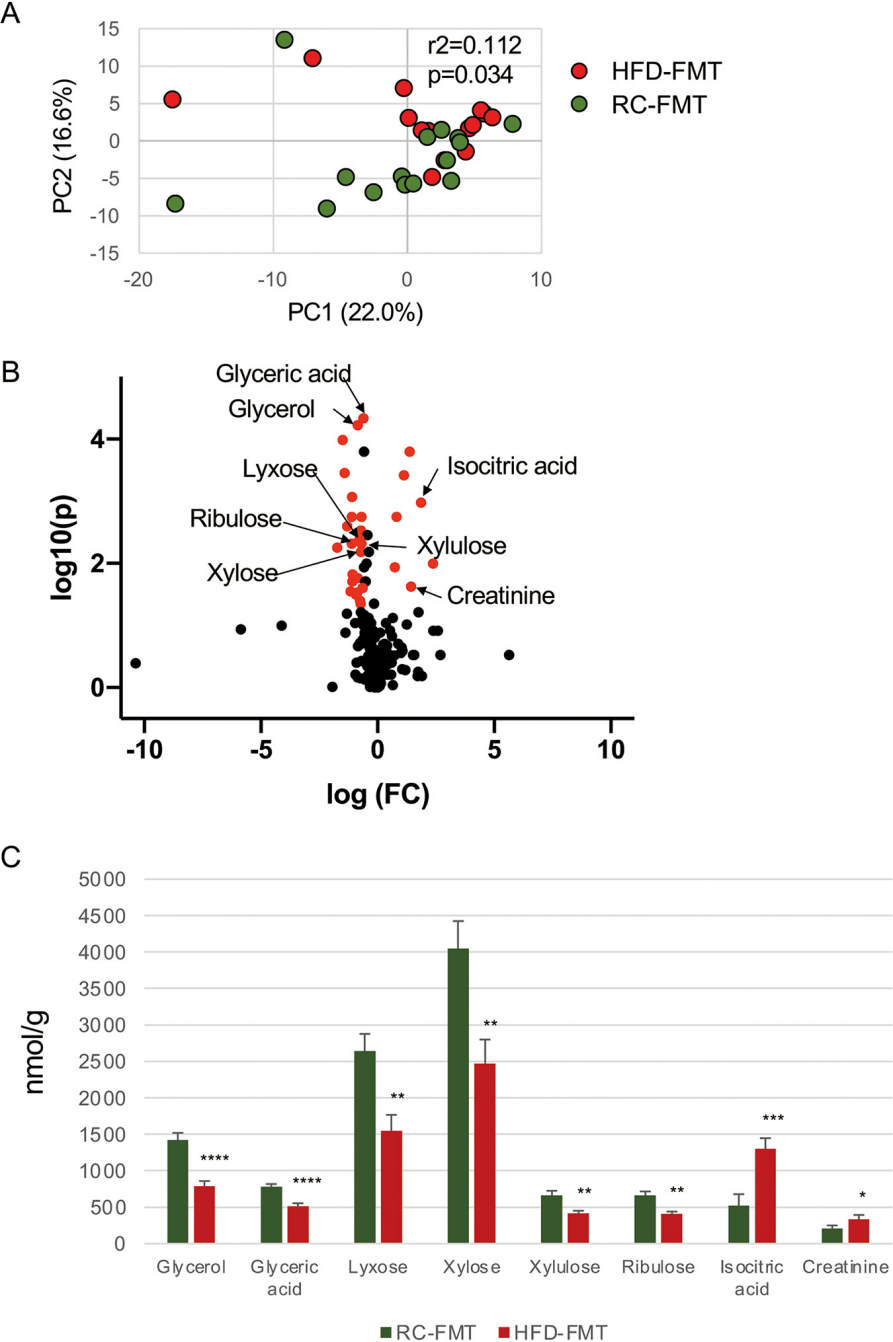
Difference in the gut metabolomic profile induced by the source of FMT. (A) Principal-component analysis (PCA) of the gut metabolomic profiles in RC-FMT (*n* = 16) and HFD-FMT (*n* = 13) mice. FMT from RC- and HFD-fed mice significantly affected recipient gut metabolomic profiles. (B) Volcano plot showing individual metabolites of RC-FMT and HFD-FMT mice. Red plots represent significantly different metabolites (fold change of >1.5 and *P* < 0.05). (C) Pairwise comparisons of significantly changed metabolites between RC-FMT and HFD-FMT mice. *, *P* < 0.05; **, *P* < 0.01; ***, *P* < 0.001; ****, *P* < 0.0001; Mann-Whitney *U*-test.

Similar to the microbiota composition, overall gut metabolomic profiles were significantly different between RC-fed (RC-donor) and HFD-fed (HFD-donor) mice (Fig. S5A). Adenine, guanosine, inosine, xanthine, and uric acid were elevated in HFD-fed mice (Fig. S5B and C), which suggested activation of the purine degradation pathway. However, the effect of FMT on the metabolomic profile of recipient mice differed from that on the microbiota composition. Although whether the gut metabolomic profile of recipient mice (RC-FMT and HFD-FMT mice) changed after FMT depended on which diet the donor mice were fed (Fig. 4A), characteristic metabolites that discriminated the two groups differed from those in donor mice. In RC-FMT mice, metabolites related to glucose metabolism (glyceric acid, glycerol, ribulose, lyxose, xylulose, and xylose) were elevated, whereas energy metabolism-related metabolites (isocitric acid and creatinine) were elevated in HFD-FMT mice (Fig. 4B and C). The differences in the gut metabolomic profile remained after 1 week irrespective of PD induction. However, the pattern of abundant metabolites was altered slightly. Nucleoside and amino acid metabolisms were higher in HFD-FMT mice than in RC-FMT mice without PD induction (Fig. S6A), and the induction of PD was associated with elevation of these metabolites in RC-FMT mice (Fig. S6B). Conversely, elevated levels of metabolites related to lipid and amino acid metabolisms were associated with the induction of PD in HFD-FMT mice (Fig. S6C). However, the metabolites were similar in RC-FMT with PD and HFD-FMT with PD mice (Fig. S6D). Thus, PD induction may affect the metabolic functions of the gut microbiota, and these effects may depend on interactions between oral and gut microbiotas.

10.1128/mBio.00771-21.5 FIG S5 Effect of RC and HFD feedings on the gut metabolomic profile. Feces from RC-fed (RC-donor) and HFD-fed (HFD donor) mice were subjected to metabolomic profiling. (A) PCA of the gut metabolomic profiles in RC donor (*n* = 6) and HFD donor (*n* = 6) mice. (B) Volcano plot showing individual metabolites in RC and HFD donor mice. (C) Pairwise comparisons of significantly changed metabolites involved in purine metabolism between RC and HFD donor mice. Red plots represent significantly different metabolites (fold change of >1.5 and *P* < 0.05). *, *P* < 0.05; **, *P* < 0.01; Mann-Whitney *U*-test. Download FIG S5, TIF file, 1.9 MB. Copyright © 2021 Sato et al. 2021 Sato et al. https://creativecommons.org/licenses/by/4.0/ This content is distributed under the terms of the Creative Commons Attribution 4.0 International license.

10.1128/mBio.00771-21.6 FIG S6 Difference in the gut metabolomic profile induced by the source of FMT and with or without periodontitis. Feces obtained after induction of periodontitis (PD) were subjected to metabolomic profiling. (A) PCA of metabolomic profiles and volcano plot showing individual metabolites in RC-FMT without PD (*n* = 8) and HFD-FMT without PD mice (*n* = 6). (B) Principal component analysis (PCA) of metabolomic profiles and volcano plot showing individual metabolites in RC-FMT without PD (*n* = 8) and with PD (*n* = 6) mice. (C) PCA of metabolomic profiles and volcano plot showing individual metabolites in HFD-FMT without PD (*n* = 6) and with PD mice (*n* = 6). (D) PCA of metabolomic profiles and volcano plot showing individual metabolites in RC-FMT with PD (*n* = 8) and HFD-FMT with PD mice (*n* = 7). Red plots represent significantly different metabolites (fold change of >1.5 and *P* < 0.05). Download FIG S6, TIF file, 1.9 MB. Copyright © 2021 Sato et al. 2021 Sato et al. https://creativecommons.org/licenses/by/4.0/ This content is distributed under the terms of the Creative Commons Attribution 4.0 International license.

### FMT and induction of PD modulate serum metabolites.

Serum metabolites associated with a change in the gut microbiome affect health and disease. To identify FMT-related factors that worsened alveolar bone resorption in ligature-induced PD, serum metabolic profiles were compared between RC-FMT and HFD-FMT mice in terms of PD.

**FIG 5:**
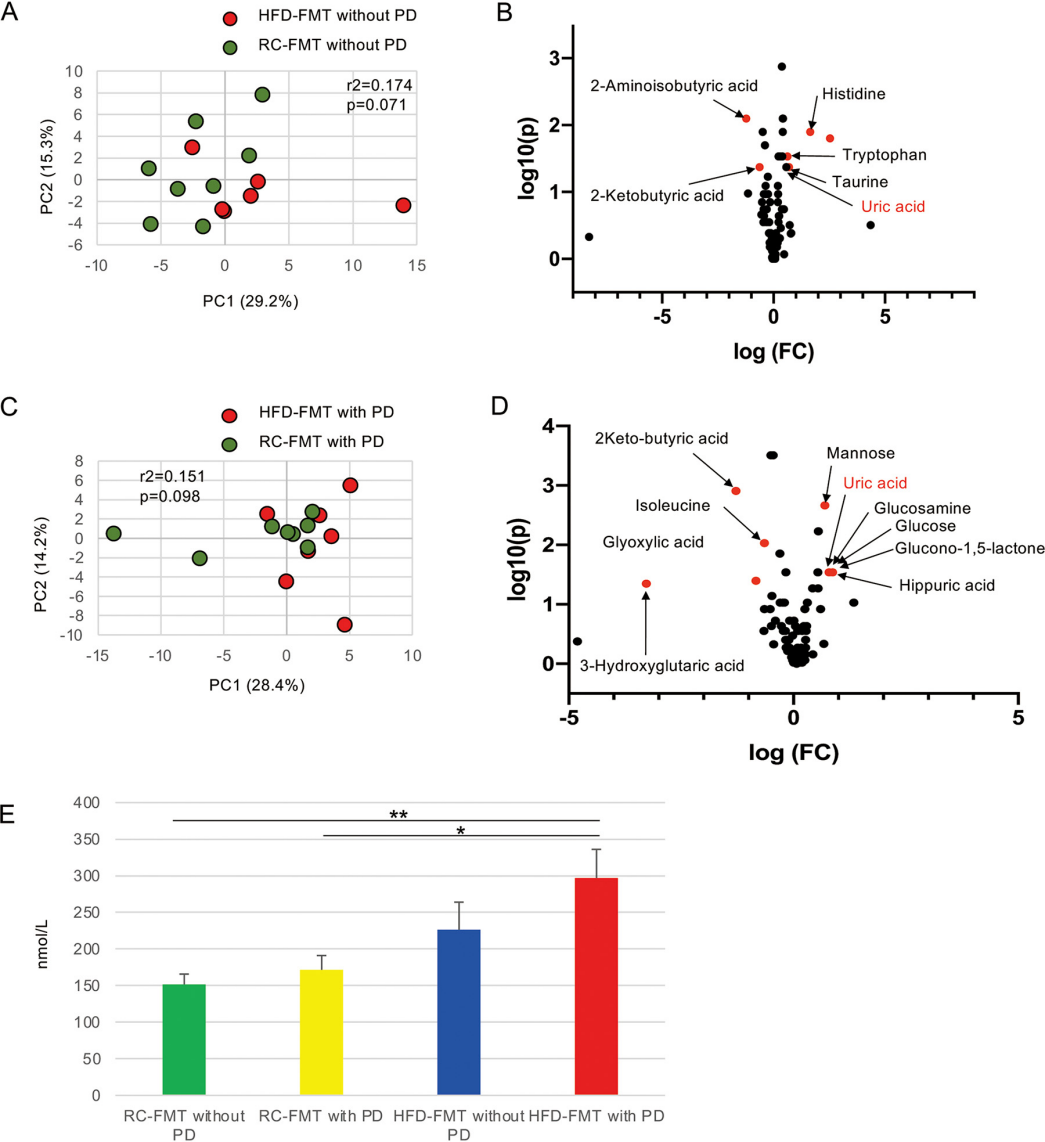
Difference in the serum metabolomic profile indued by the source of FMT and induction of periodontitis (PD). (A) PCA of the serum metabolomic profiles of RC-FMT without PD (*n* = 8) and HFD-FMT without PD (*n* = 6) mice. (B) Volcano plot showing individual serum metabolites in RC-FMT without PD and HFD-FMT without PD mice. (C) PCA of the serum metabolomic profiles of RC-FMT with PD (*n* = 8) and HFD-FMT with PD (*n* = 7) mice. (D) Volcano plot showing individual serum metabolites in RC-FMT with PD and HFD-FMT with PD mice. Red plots represent significantly different metabolites (fold change of >1.5 and *P* < 0.05). (E) Comparison of serum levels of uric acid after FMT and with or without induction of PD. *, *P* < 0.05; **, *P* < 0.01; Mann-Whitney *U*-test or one-way ANOVA with Bonferroni’s correction for multiple comparisons.

The source of FMT slightly affected serum metabolomic profiles without induction of PD (Fig. 5A) with a significant change in the relative abundance of amino acid metabolites (Fig. 5B). Additionally, the presence or absence of periodontitis had little effect on the serum metabolomic profile irrespective of the source of FMT (Fig. S7A and B). Although the effect of the FMT source on serum metabolites was not very obvious even after induction of PD (Fig. 5C and D), uric acid was of particular interest among the metabolites that differed between the two groups, because it is reportedly elevated in PD patients (9). In fact, the serum level of uric acid was higher in HFD-FMT mice compared with RC-FMT mice, with a significant difference when PD was induced (Fig. 5B and D). Interestingly, the serum uric acid level was increased with a greater burden of systemic and local insults (Fig. 5E). However, no difference was observed in fecal uric acid (Fig. S8).

10.1128/mBio.00771-21.7 FIG S7 Difference in the serum metabolomic profile induced by the source of FMT. Sera were obtained from mice in which RC-FMT and HFD-FMT mice had received fecal microbiota of RC- or HFD-fed mice, respectively. (A) PCA of the serum metabolomic profiles and volcano plot showing individual serum metabolites in RC-FMT without PD (*n* = 8) and with PD (*n* = 8) mice. (B) PCA of the serum metabolomic profiles and volcano plot showing individual serum metabolites in HFD-FMT without PD (*n* = 6) and with PD (*n* = 7) mice. Red plots represent significantly different metabolites (fold change of >1.5 and *P* < 0.05). Download FIG S7, TIF file, 1.9 MB. Copyright © 2021 Sato et al. 2021 Sato et al. https://creativecommons.org/licenses/by/4.0/ This content is distributed under the terms of the Creative Commons Attribution 4.0 International license.

10.1128/mBio.00771-21.8 FIG S8 Comparison of fecal uric acid levels after FMT and with or without induction of periodontitis. Feces obtained at the end of the experimental period were analyzed. Download FIG S8, TIF file, 1.9 MB. Copyright © 2021 Sato et al. 2021 Sato et al. https://creativecommons.org/licenses/by/4.0/ This content is distributed under the terms of the Creative Commons Attribution 4.0 International license.

### Uric acid worsens bone destruction.

**FIG 6:**
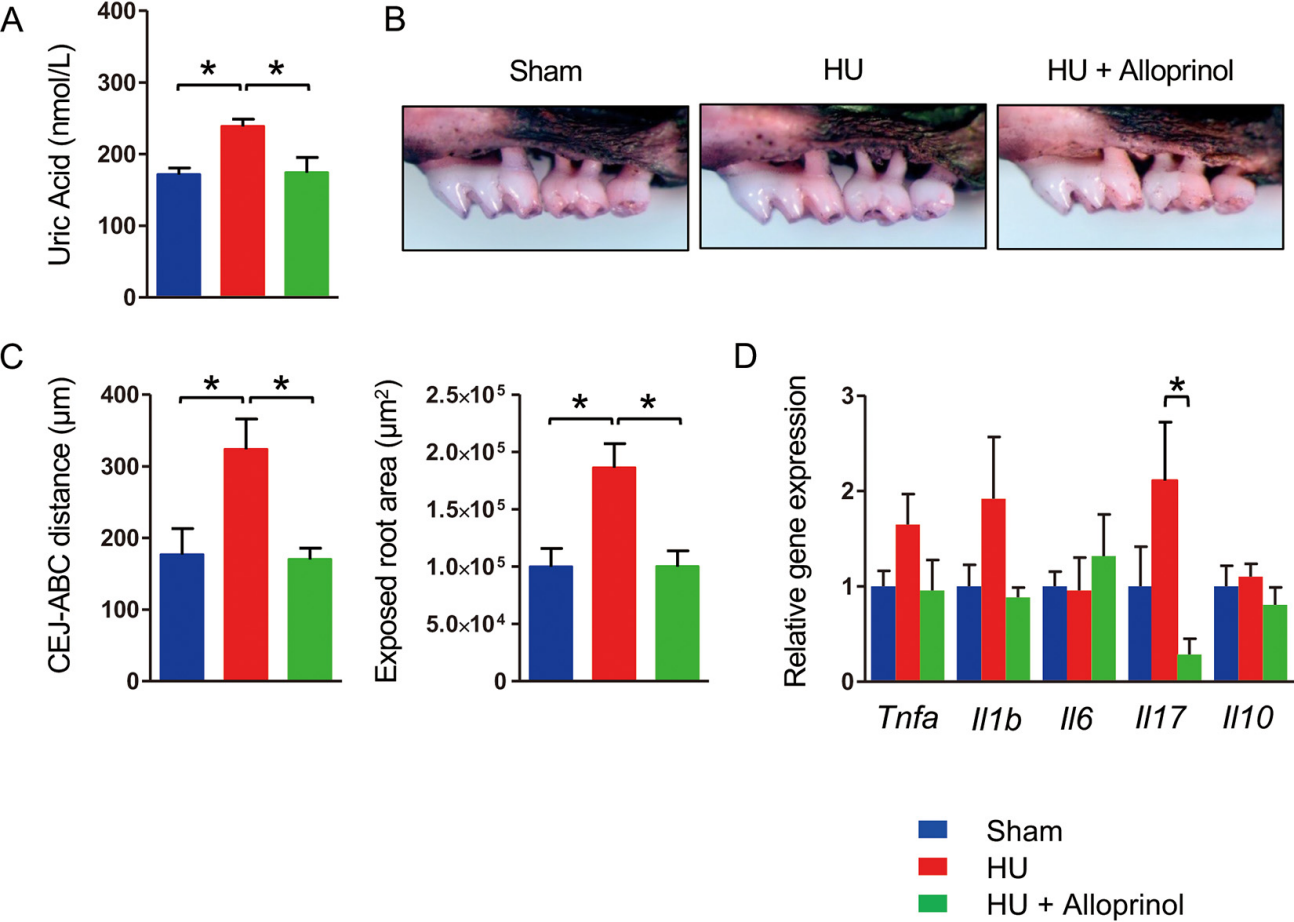
Hyperuricemia (HU) aggravates ligature-induced periodontitis. Three days after ligature placement, C57BL/6N mice were administered PBS, uric acid (125 mg/kg), or uric acid plus allopurinol (5 mg/kg) once a day for 4 days. PBS and uric acid were administered intraperitoneally, and allopurinol was administered via gastric gavage (*n* = 4 for uric acid and uric acid plus allopurinol, *n* = 3 for PBS). (A) Serum uric acid level in each group. Data are expressed as the mean ± SEM. (B) Effects of hyperuricemia on alveolar bone resorption. Representative images obtained after soft tissue removal are shown. (C) The distance between the cementoenamel junction and alveolar bone crest and the exposed tooth root area of the mesial root of the maxillary second molar was measured under a stereoscopic microscope. (D) Relative gene expression levels in the gingiva of each experimental group. The relative quantity of mRNA was normalized to that of glyceraldehyde-3-phosphate dehydrogenase mRNA. *, *P* < 0.05; one-way ANOVA with Bonferroni’s correction for multiple comparisons.

We hypothesized that an elevated uric acid level was associated with deterioration of ligature-induced PD. To test this hypothesis, we induced PD in hyperuricemic mice by administering uric acid intraperitoneally. Administration of uric acid significantly elevated the serum uric acid level comparably with that seen in HFD-FMT mice with periodontitis (Fig. 6A). Weak bone destruction was observed in sham-administered mice. However, severe bone destruction was seen in uric acid-administered mice (Fig. 6B). A significantly greater distance between the cementoenamel junction and alveolar bone crest and an exposed tooth root surface area were noted (Fig. 6C). Concomitant administration of allopurinol, a xanthine oxidase inhibitor, completely suppressed elevation of the serum uric acid level and abrogated the worsened uric acid-associated bone destruction. Among the inflammatory cytokine genes in gingiva, expression of *Tnfa* and *Il1b* tended to be higher in hyperuricemic mice than in the other groups (Fig. 6D).

**FIG 7:**
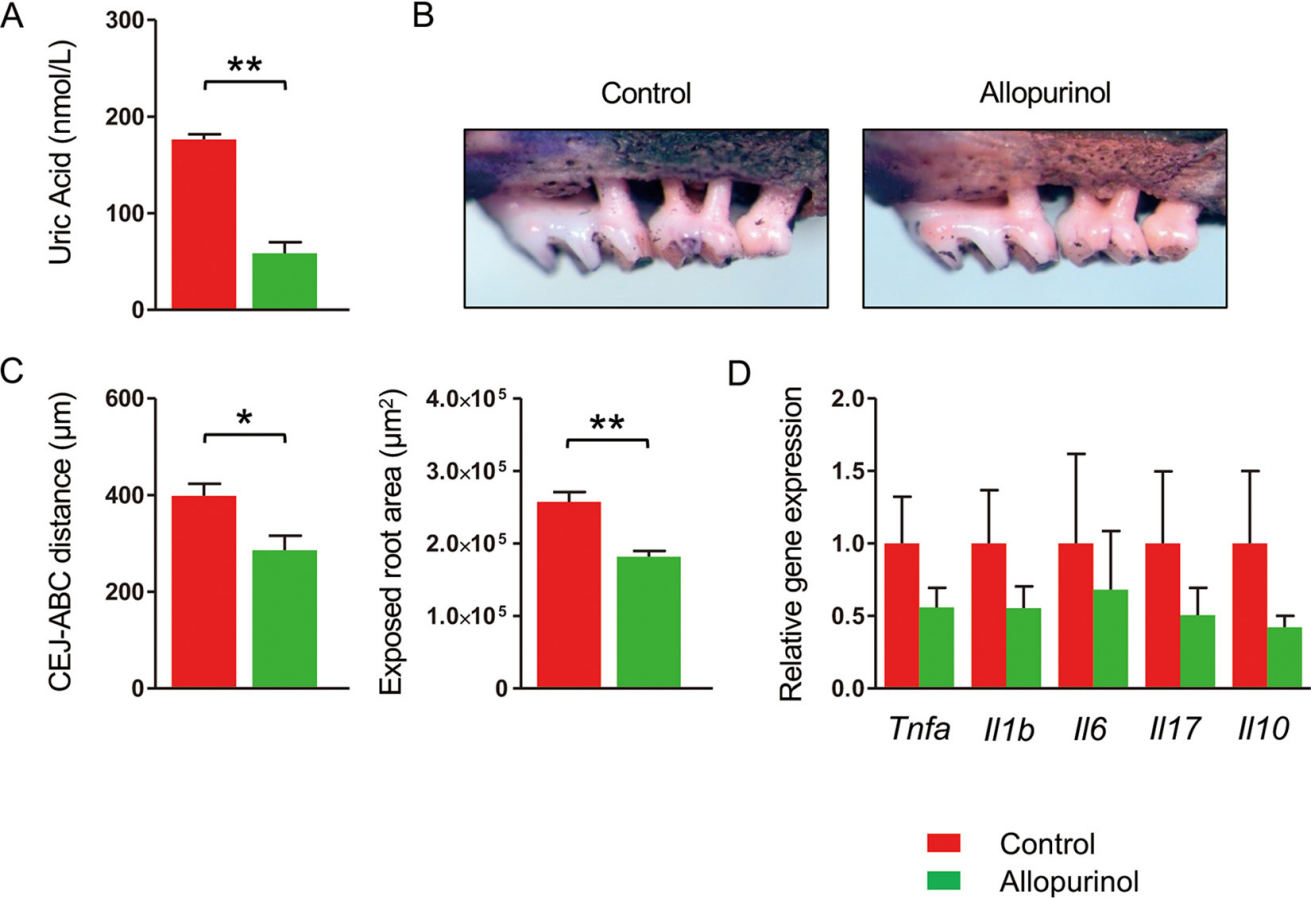
Allopurinol suppresses experimental periodontitis (PD) in HFD-FMT mice. Mice that received FMT from high-fat diet-fed mice were subjected to ligature-induced PD for 1 week. The mice were administered either allopurinol (5 mg/kg) (*n* = 6) or PBS (*n* = 6) every other day during the PD period. (A) Serum uric acid level in each group. (B) Effects of allopurinol on alveolar bone resorption. Representative images obtained after soft tissue removal are shown. (C) The distances between the cementoenamel junction and alveolar bone crest and the exposed tooth root area of the mesial root of the maxillary second molar were measured under a stereoscopic microscope. (D) Relative gene expression levels in the gingiva of each experimental group. The relative quantity of mRNA was normalized to that of glyceraldehyde-3-phosphate dehydrogenase mRNA. Data are expressed as the mean ± SEM. *, *P* < 0.05; **, *P* < 0.01; Mann-Whitney *U*-test.

To confirm the involvement of uric acid in the elevated alveolar bone resorption, we administered allopurinol to HFD-FMT with PD mice. Allopurinol administration significantly decreased the serum uric acid level (Fig. 7A) and significantly suppressed the bone destruction compared with PBS-administered mice (Fig. 7B and C), although the expression of inflammatory cytokines tended to be lower in allopurinol-administered mice (Fig. 7D). These results confirmed that uric acid is responsible for obesity-related worsening of PD.

## DISCUSSION

Complex interactions between dysbiosis of the oral microflora and host responses to bacterial insult underlie the etiopathogenesis of PD. However, epidemiological studies have demonstrated that obesity also contributes to initiation and progression of the disease. The association between obesity and PD is consistent with a compelling pattern of increased risk of PD in overweight or obese individuals. Although an exaggerated host immune response, altered periodontal microflora, insulin resistance, increased proinflammatory cytokines, and oxidative stress are hypothetical mechanisms that connect obesity and PD, the precise mechanisms remain unclear (1).

PD is associated with an increased risk of type 2 diabetes (10), cardiovascular disease (11), and chronic kidney disease (12), which are well-known comorbidities of obesity. Recent evidence suggests that alterations in the gut microbiome predispose to the development of these diseases. We demonstrated that P. gingivalis, a representative periodontopathic bacterium, induces gut dysbiosis (13–15) and modulates the disease (16, 17). The role of the gut microbiota in obesity is well documented in animal and human studies despite inconsistent results of individual studies. Thus, the relationship is far more complex than initially thought (18). Nevertheless, considering the interrelationship between PD and obesity from a gut microbial viewpoint, gut dysbiosis appears to play a negative role in PD.

We used FMT to provide the first direct evidence of the role of the gut microbiota in obesity as a risk factor for PD. FMT was conducted by direct administration of gut microbiota into the stomach using a feeding needle. Because FMT and ligature placement had little effect on the oral microflora, the effect of FMT on the oral microbiota was excluded. Furthermore, our metabolomic analysis demonstrated that uric acid may be a critical molecule in obesity-associated PD.

Although direct evidence of a negative effect of gut dysbiosis on the pathogenesis of PD has been lacking, several studies have unintentionally provided implied circumstantial evidence. An HFD exacerbates lipopolysaccharide-induced or -associated PD in mice (19, 20). Feeding a fish oil-supplemented, but not corn oil-supplemented, diet, which contained high levels of omega-3 and -6 fatty acids, respectively, suppresses P. gingivalis-induced PD in rats (5). Omega-3 fatty acids have beneficial effects on the gut microbiota (4). Probiotic (B. subtilis) supplementation in drinking water reduces ligature-induced periodontal tissue destruction and improves intestinal morphology in rats (6). Additionally, ligature-induced PD decreases in rats after oral administration of metformin, an oral drug for type 2 diabetes (21). Although metformin suppresses the inflammatory response by activating AMP-activated protein kinase (22), recent evidence shows that the effect of metformin on the blood glucose level depends on modulation of the gut microbiota (7, 23). Our results corroborate the influence of the gut microbiota on the periodontal condition.

Uric acid is a product of metabolic breakdown of purine nucleotides derived from both exogenous dietary and endogenous nucleic acids. While a certain level of uric acid is thought to be beneficial as an antioxidant, excessive uric acid is associated with several disorders, including gout, cardiovascular disease, and kidney stone formation. Several epidemiological studies have demonstrated a positive correlation between obesity and elevated blood uric acid levels. In particular, visceral adiposity is a major contributor to elevated uric acid. Furthermore, weight loss from bariatric surgery is associated with a reduced incidence of hyperuricemia (24).

Some observational human and animal studies have shown elevated uric acid levels in the presence of PD with or without comorbidities (9). An interventional study also showed a reduction in serum uric acid levels after nonsurgical periodontal therapy (25). However, other studies have shown no change or a reversal of blood uric acid levels in PD patients (26, 27). Thus, while several studies have examined the effect of PD on systemic uric acid levels, to our knowledge, this is the first study to demonstrate the role of uric acid in PD pathology.

In contrast to uric acid in systemic circulation, salivary uric acid levels are frequently downregulated in PD patients and increase after treatment. Although the link between decreased uric acid levels in PD patients and greater disease severity has been described in the context of lower antioxidant properties (28), the contradictory results between serum and salivary uric acid levels could be caused by oral bacteria in PD patients.

The gut microbiota is involved in purine metabolism and uric acid production. However, little is known about its effect on host purine metabolism. Transplantation of gut microbiota from hyperuricemic rats increases serum uric acid in recipient rats (29). Additionally, several taxa of gut microbiota are reportedly associated with elevated serum uric acid levels in obese subjects (30). Colonization of Saccharomyces cerevisiae, a member of the gut microbes, enhances host purine metabolism, which increases the serum uric acid level (31). However, bacterial taxa that enhance host purine metabolism have not been identified, despite reports of hyperuricemia-associated bacteria (29, 30). We identified transfer of *Enterococcus* and *Akkermansia* from HFD donors to HFD-FMT mice, whereas Unc. S24-7 and Unc. *Erysipelotrichaceae* were transferred from RC donors to RC-FMT mice. Although the role of these bacteria in host purine metabolism is unknown, it is interesting that *Akkermansia* and *Turicibacter* have a purine metabolism pathway that potentially elevates uric acid synthesis (Kyoto Encyclopedia of Genes and Genomes pathway). Additionally, elevated *Turicibacter* is reported to be associated with hyperuricemia (32). Although these findings imply that *Akkermansia*, *Turicibacter*, and *Enterococcus* are involved in the elevation of uric acid, their causal effects are not known. Conversely, HFD feeding induced a decrease in the relative abundance of *Lactobacillus*. Many *Lactobacilli* are auxotrophic for purines and pyrimidines, and some strains have an additional requirement of deoxynucleoside for DNA synthesis (33). Therefore, a decrease of *Lactobacillus* may increase purine incorporation from the gut. Thus, FMT from HFD donor mice induced recipient mice to be prone to hyperuricemia. Nevertheless, correlation analysis did not show involvement of particular microbes in any metabolite related to the purine metabolism pathway (data not shown).

The mechanism by which uric acid aggravates alveolar bone destruction can be explained by enhancement of proinflammatory cytokines (34, 35), oxidative stress (36, 37), and pathological bone remodeling (38, 39). We demonstrated that direct induction of hyperuricemia by intraperitoneal administration of uric acid caused more severe alveolar bone resorption in mice with ligature-induced PD than in phosphate-buffered saline-administered mice and that the detrimental effect of uric acid was completely suppressed by concomitant administration of allopurinol. Additionally, administration of allopurinol to HFD-FMT mice with PD induced significant suppression of alveolar bone resorption compared with PBS administration. Although allopurinol has no effect on synthesized uric acid, uric acid has been demonstrated to stimulate IL-1β production via NLRP3 inflammasome activation (40), which in turn activates xanthine oxidase (41). Therefore, it is conceivable that administered allopurinol inhibited secondary production of uric acid. Nevertheless, our results suggest an unequivocal effect of uric acid on bone destruction.

Transplantation of fecal microbiota in HFD-fed mice activated the purine degradation pathway and elevated serum uric acid levels with significant elevation upon induction of PD. This suggests that a change in the oral microbiota and/or periodontal inflammation induced by ligature placement had some effect on the systemic purine degradation pathway because of the difference in the oral microbiota between HFD-FMT mice with or without PD.

Thus, uric acid may deteriorate PD in obese patients. However, little is known about the complex interactions between gut dysbiosis and the serum metabolomic profile. Further studies are warranted to clarify the etiopathogenesis of obesity in patients with inflammatory bone destruction, particularly obese patients with PD.

## MATERIALS AND METHODS

### Experimental model.

Four-week-old male C57BL/6N mice were obtained from Japan SLC (Shizuoka, Japan). The mice were acclimatized under specific-pathogen-free conditions and fed regular chow (CE2; CLEA Japan, Tokyo, Japan) and sterile water for 1 week. The mice were then divided into a regular chow (RC) group (fed CE2) and high-fat diet (HFD) group (fed HFD32; CLEA Japan). Feces were collected at 4, 6, and 8 weeks after the diet change, subjected to 16S rRNA gene sequencing, and stored at −80°C until use. A 100 mg aliquot of mixed fecal samples was suspended in 1 ml phosphate-buffered saline (PBS) and vortexed for 10 s. After centrifugation at 800 × *g* for 3 min, the supernatant was collected for fecal microbial transplantation (FMT).

For the transplantation experiment, 6-week-old male C57BL/6N mice purchased from Japan SLC were acclimatized for 1 week and then received an antibiotic cocktail (1 g/liter ampicillin, 1 g/liter neomycin, 1 g/liter metronidazole, and 500 mg/liter vancomycin) in water *ad libitum* for 1 week. After antibiotic treatment, 200 μl of a fecal sample from RC- and HFD-fed mice was inoculated via gastric gavage (disposable feeding needle; Kenis Ltd., Osaka, Japan) once a day for 5 consecutive days (here referred to as RC-FMT and HFD-FMT mice, respectively). PBS-administered mice served as controls. Mice from each group were sacrificed to analyze the effect of FMT alone. One mouse in the RC-FMT group died on the fourth day of FMT.

For the combined FMT and experimental periodontitis (PD) experiment, ligature placement with 5-0 silk was conducted around the bilateral maxillary second molar, followed by the above-described FMT procedure (here referred to as RC-FMT with PD and HFD-FMT with PD). The ligature was left in place for 1 week. Animals that received FMT and were maintained for the same period without ligation served as controls (referred to as RC-FMT without PD and HFD-FMT without PD) (Fig. S1B). Some HFD-FMT mice with induction of PD received allopurinol administration. One mouse in the HFD-FMT with PD group and two mice in the HFD-FMT group died during fecal transplantation.

Ligature-induced experimental PD was induced in 8-week-old male C57BL/6N mice (Japan SLC) as described above. From day 3 after ligature placement, the mice were administered PBS, uric acid (125 mg/kg), or uric acid plus allopurinol (5 mg/kg) once a day for 4 consecutive days. PBS and uric acid were administered intraperitoneally, and allopurinol was administered via gastric gavage.

This study was approved by the Institutional Animal Care and Use Committee at Niigata University (permit numbers SA00472 and SA00634). All experiments were performed in accordance with the Regulations and Guidelines on Scientific and Ethical Care and Use of Laboratory Animals of the Science Council of Japan, enforced on 1 June 2006.

### Sample collection.

Samples of oral bacteria were collected by swabbing the surfaces of soft and hard oral tissues with three sterile number 40 paper points (Zipperer absorbent paper points; VDW GmbH, Munich, Germany) after FMT (RC-FMT and HFD-FMT groups) and at the end of the experiments. Feces were collected at three time points—after antibiotic treatment, after FMT, and after the ligature period. Sera for metabolomics were obtained after FMT and the ligature period. Gingival tissue was obtained from the ligated tooth after the ligature period.

Intestinal tissues, mesenteric lymph nodes, and blood were obtained before induction of periodontitis to analyze the effect of FMT alone.

### Periodontal tissue histology.

The fixed maxillae of two mice from each ligated group were dissected, decalcified, embedded, and sectioned as described previously (42). Serial 5-μm-thick sections were obtained in the sagittal direction along the long axis of teeth. The sections were stained with hematoxylin and then imaged under a microscope (Biozero BZ-8000; Keyence, Tokyo, Japan).

### Quantification of alveolar bone loss.

The amount of bone loss was assessed in images obtained under a stereomicroscope fitted with a video image marker measurement system (DP2-BSW; Olympus, Tokyo, Japan).

The distance from the alveolar bone crest to the cementoenamel junction of the maxillary second molar was analyzed on the mesial root. The measurement of alveolar bone loss was performed by one examiner (K.Y.) in a blinded manner. Measurements were repeated three times, and the level of agreement was 0.9989.

### DNA extraction from oral and fecal samples.

Fecal and salivary samples were collected and stored at −80°C until analysis. Fecal pellets were suspended in 450 μl Tris-EDTA (TE) buffer. DNA extraction from oral bacteria was performed after soaking paper points in TE buffer for 2 h. The bacterial suspension was incubated with 15 mg/ml lysozyme (Fujifilm Wako Pure Chemical Corporation, Osaka, Japan) at 37°C for 1 h. Purified achromopeptidase (Fujifilm Wako Pure Chemical Corporation) was added at a final concentration of 2,000 U/ml, followed by incubation at 37°C for 30 min. The suspension was mixed with 1% (wt/vol) sodium dodecyl sulfate and 1 mg/ml proteinase K (Merck, Darmstadt, Germany) and incubated at 55°C for 1 h. After centrifugation, the bacterial DNA was purified using a phenol-chloroform/isoamyl alcohol (25:24:1) solution. The DNA was precipitated by adding ethanol and 3 M sodium acetate. RNase treatment and polyethylene glycol precipitation were performed. The DNA pellet was dried and then dissolved in TE buffer.

### 16S rRNA gene sequencing.

The V4 variable region (515F-806R) of samples was sequenced using Illumina Miseq following the method of Kozich et al. (43). Primers with adaptor sequences for the Illumina Miseq platform were as follows: forward primer, 5′-AATGATACGGCGACCACCGAGATCTACAC NNNNNNNN TATGGTAATTGTGTGCCAGCMGCCGCGGTAA-3′′; reverse primer, 5′-CAAGCAGAAGACGGCATACGAGAT NNNNNNNN AGTCAGTCAGCCGGACTACHVGGGTWTCTAAT-3′. The “NNNNNNNN” sequence unique to each sample was attached to the primer for multiplexing. The PCR mixture contained 15 pmol of each primer, 50 ng microbial DNA, 4 μl of 2 mM deoxynucleoside triphosphate (dNTP) mixture, 5 μl 10× *Ex Taq* buffer, 0.25 μl *Ex Taq* Hot Start version (TaKaRa Bio, Inc., Shiga, Japan), and sterile water to reach a final volume of 50 μl.

PCR conditions were as follows: 95°C for 2 min and then 25 cycles of 95°C for 30 sec, 55°C for 15 sec, and 72°C for 1 min, followed by 72°C for 3 min. The PCR products were purified using AMPure XP (Beckman Coulter, Brea, CA, USA) and quantified using a Quant-iT PicoGreen double-stranded DNA (dsDNA) assay kit (Life Technologies Japan, Tokyo, Japan). Mixed samples were prepared by pooling approximately equal amounts of PCR amplicons from each sample. The pooled library was analyzed using a TapeStation high-sensitivity DNA 1000 assay (Agilent Technologies, Santa Clara, CA, USA). Real-time PCR quantification of the pooled library was performed using a NEBNext Library Quant kit for Illumina (New England Biolabs Japan, Inc., Tokyo, Japan), which followed the manufacturer’s protocols.

On the basis of the quantification, the sample library was denatured and diluted. A DNA library that contained 20% denatured PhiX spike-in was sequenced with Miseq technology using a 500-cycle kit (Illumina, Inc., San Diego, CA, USA). The sequence data reported in this study have been deposited in the DNA Data Bank of Japan under accession numbers DRA010642 and DRA010643 (www.ddbj.nig.ac.jp).

### Metabolome analysis.

The following were added to 10-μl aliquots of serum: 150 μl methanol, 125 μl Milli-Q water, 15 μl internal standard solution (1 mmol/liter 2-isopropylmalic acid), and 60 μl chloroform. The following were added to 5-mg samples of feces: 150 μl methanol, 150 μl Milli-Q water containing internal standard (100 μmol/liter 2-isopropylmalic acid), and 60 μl chloroform. The solution was shaken at 1,200 rpm for 30 min at 37°C. After centrifugation at 16,000 × *g* for 5 min at room temperature, 250 μl of the supernatant was transferred to a new tube, and 200 μl Milli-Q water was added. The solution was mixed and then centrifuged at 16,000 × *g* for 5 min at room temperature, and 250 μl of the supernatant was transferred to a new tube. Samples were dried using a vacuum evaporator for 20 min at 40°C and lyophilized using a freeze dryer. Dried extracts were first methoxylated with 40 μl of 20 mg/ml methoxyamine hydrochloride (Sigma-Aldrich, St. Louis, MO, USA) dissolved in pyridine and shaken at 1,200 rpm for 90 min at 30°C. The solution was then silylated with 20 μl *N*-methyl-*N*-(trimethylsilyl)trifluoroacetamide (GL Sciences, Tokyo, Japan) for 30 min at 37°C with shaking at 1,200 rpm. After derivatization, the samples were centrifuged at 16,000 × *g* for 5 min at room temperature, and the supernatant was transferred to a glass vial. Analysis was performed using a gas chromatography (GC)-tandem mass spectrometry platform on a triple quadrupole mass spectrometer (GCMS-TQ8030; Shimadzu, Kyoto, Japan) with a capillary column (BPX5; SGE Analytical Science/Trajan Scientific and Medical, Ringwood, Victoria, Australia). The GC oven was programmed as follows: 60°C for 2 min, increased to 330°C (15°C/min), and finally 330°C for 3.45 min. GC was operated in constant linear velocity mode set to 39 cm/s. The detector and injector temperatures were 200°C and 250°C, respectively. The injection volume was set at 1 μl with a split ratio of 1:30.

### qPCR.

Total RNA was isolated using TRI Reagent (Molecular Research Center, Inc., Cincinnati, OH, USA) in accordance with the manufacturer’s instructions. cDNA was synthesized with Transcriptor Universal cDNA Master (Roche Molecular Systems, Pleasanton, CA, USA). FastStart Essential DNA Green Master (Roche Molecular Systems) was used to amplify mRNA in a LightCycler 96 instrument (Roche Molecular Systems) for quantitative PCR analysis. The expression of each mRNA was normalized to GAPDH (glyceraldehyde-3-phosphate dehydrogenase) using the ΔΔ*CT* method. The oligonucleotide sequences (Thermo Fisher Scientific, Waltham, MA, USA) used in this study are listed in Table S1.

10.1128/mBio.00771-21.9 TABLE S1 Sequences of primers used for qPCR. Download Table S1, DOCX file, 0.02 MB. Copyright © 2021 Sato et al. 2021 Sato et al. https://creativecommons.org/licenses/by/4.0/ This content is distributed under the terms of the Creative Commons Attribution 4.0 International license.

### Flow cytometry.

To analyze intracellular expression of IL-17 and Foxp3, cells from mesenteric and submandibular lymph nodes were adjusted to a concentration of 1 × 10^6^/ml in RPMI 1640 (Sigma-Aldrich) supplemented with 10% fetal bovine serum (Sigma-Aldrich) and then stimulated with 50 ng/ml phorbol myristate acetate (PMA) (Sigma-Aldrich) and 1 μg/ml ionomycin (Calbiochem, San Diego, CA, USA) in the same medium for 24 h. BD GolgiPlug (BD Biosciences, San Jose, CA, USA) was added 16 h after the start of incubation. After harvesting the cells by centrifugation, a Cytofix/Cytoperm Plus fixation/permeabilization kit (BD Biosciences) was used for staining with a fluorescein isothiocyanate (FITC)-labeled anti-IL-17A or phycoerythrin (PE)-labeled anti-Foxp3 antibody and a specific peridinin chlorophyll protein (PerCP)-labeled anti-CD4 antibody (eBioscience, San Diego, CA, USA) in accordance with the manufacturer’s instructions. The expression level of each molecule was analyzed using a FACSAria II instrument and FlowJo (TOMY Digital Biology, Tokyo, Japan).

### Uric acid measurement.

The serum level of uric acid was determined using a uric acid assay kit (ab65344; Abcam, Cambridge, UK) in accordance with the manufacturer’s instructions.

### Bioinformatics and statistical analyses.

Taxonomic assignments and estimation of relative abundance from sequencing data were performed using the analysis pipeline of QIIME version 1.9.1 (44). An operational taxonomic unit (OTU) was defined at 97% similarity using UCLUST (45, 46). OTU taxonomy was assigned on the basis of comparison with the Greengenes database version 13.8 (47). β-Diversity was calculated using weighted UniFrac distances on the basis of the OTU distribution across samples and visualized by principal-coordinate analysis (PCoA). The quantified metabolome data were normalized by an autoscaling method and statistically analyzed using principal-component analysis (PCA).

Statistical analyses were performed using Prism version 9 (GraphPad Software, Inc., La Jolla, CA, USA) and R version 4.0.4. Neither randomization nor blinding was performed in this study. All data are expressed as the mean ± standard error of the mean. To compare the data of two groups, the differences were evaluated using the Mann-Whitney *U*-test. For comparisons of more than three groups, statistical analyses were performed using one-way analysis of variance with Bonferroni’s correction. Analysis of similarity was performed to identify differences in bacterial community compositions, and PERMANOVA (permutational multivariant analysis of variance) was used for comparison of microbes between groups. *P* values of <0.05 were considered statistically significant.
