# Supplementary figures and images for: Obesity-Related Gut Microbiota Aggravates Alveolar Bone Destruction in Experimental Periodontitis through Elevation of Uric Acid
Source: mBio. 2021 Jun 1;12(3):e00771-21. doi: 10.1128/mBio.00771-21 (PMC8262938; doi:10.1128/mBio.00771-21)

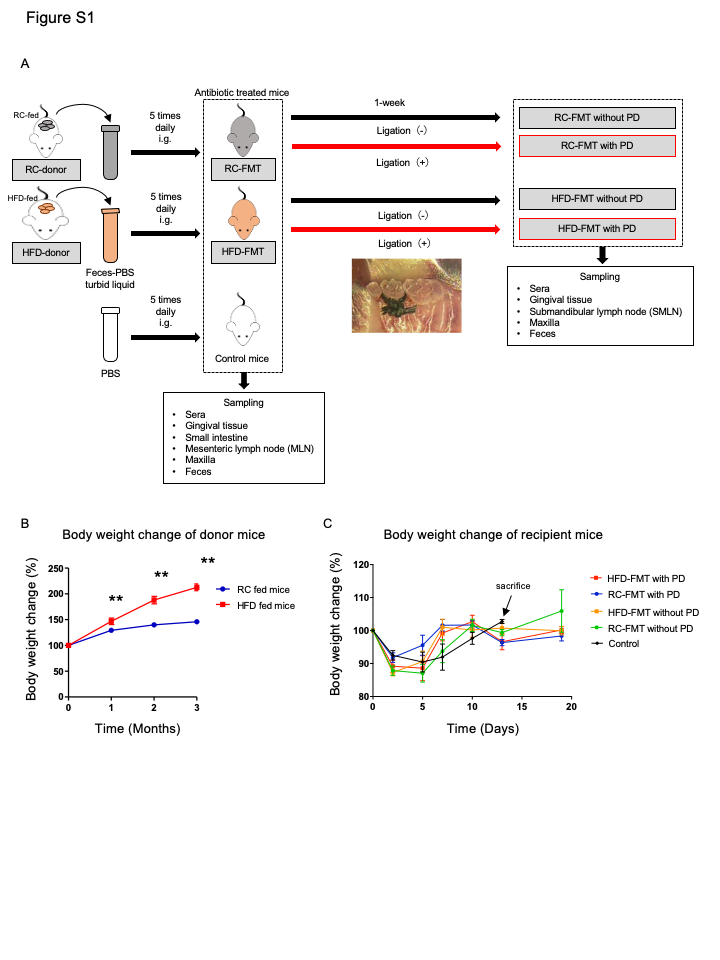

Supplement: FIG S1 [file mbio.00771-21-sf001.tif]

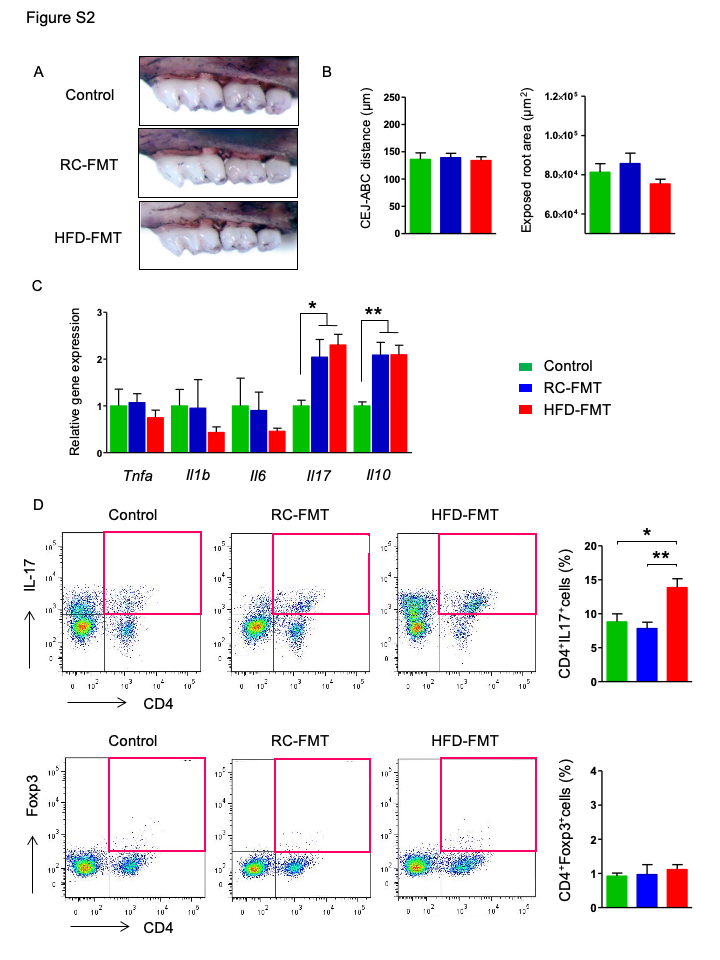

Supplement: FIG S2 [file mbio.00771-21-sf002.tif]

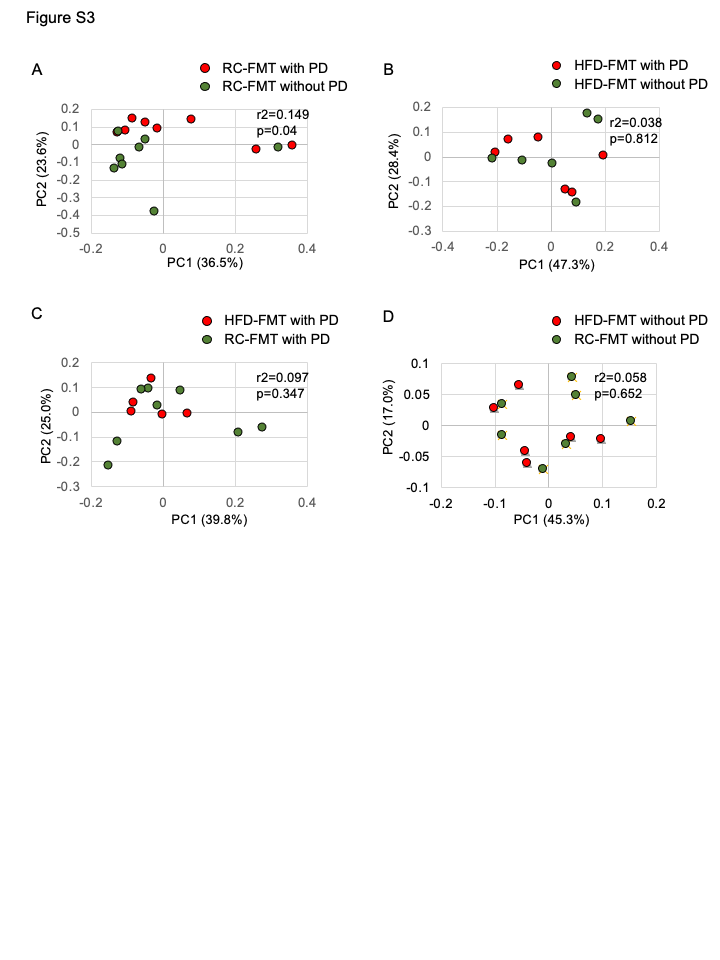

Supplement: FIG S3 [file mbio.00771-21-sf003.tif]

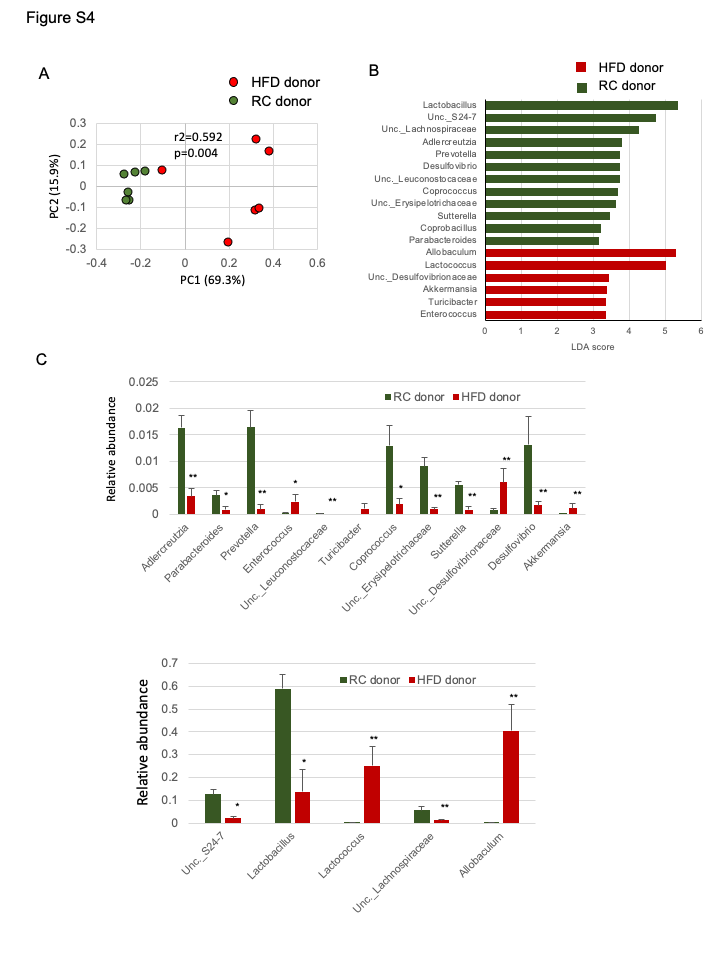

Supplement: FIG S4 [file mbio.00771-21-sf004.tif]

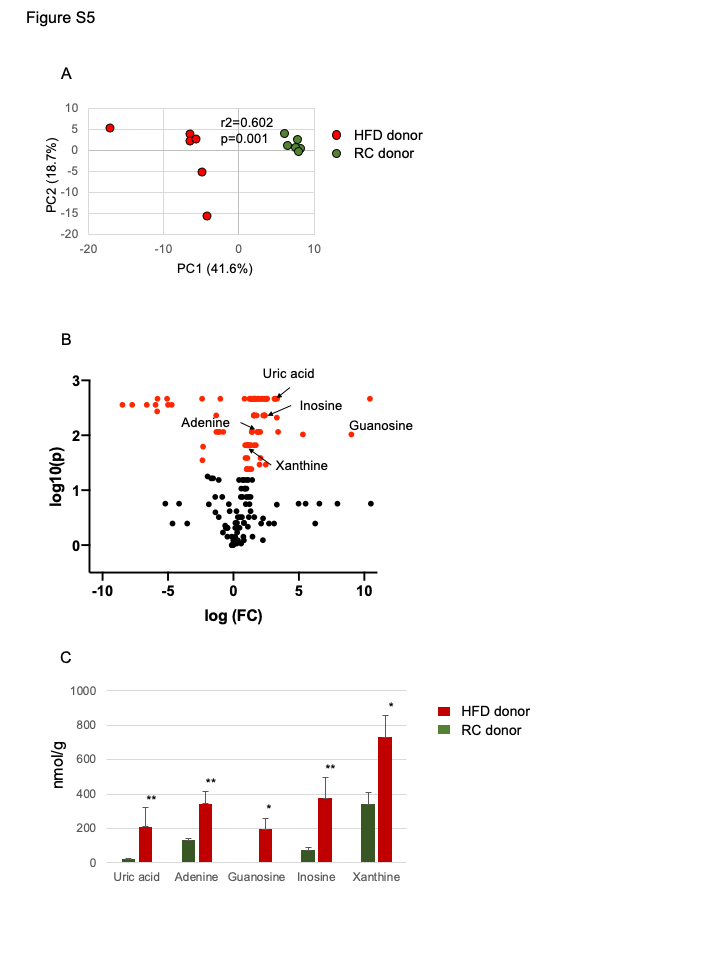

Supplement: FIG S5 [file mbio.00771-21-sf005.tif]

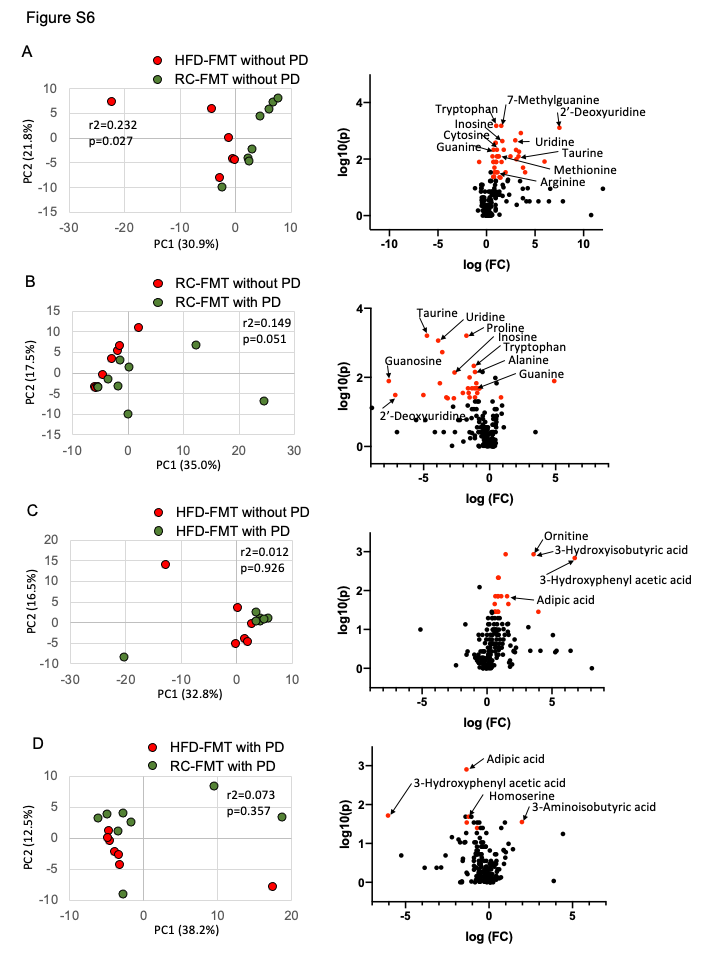

Supplement: FIG S6 [file mbio.00771-21-sf006.tif]

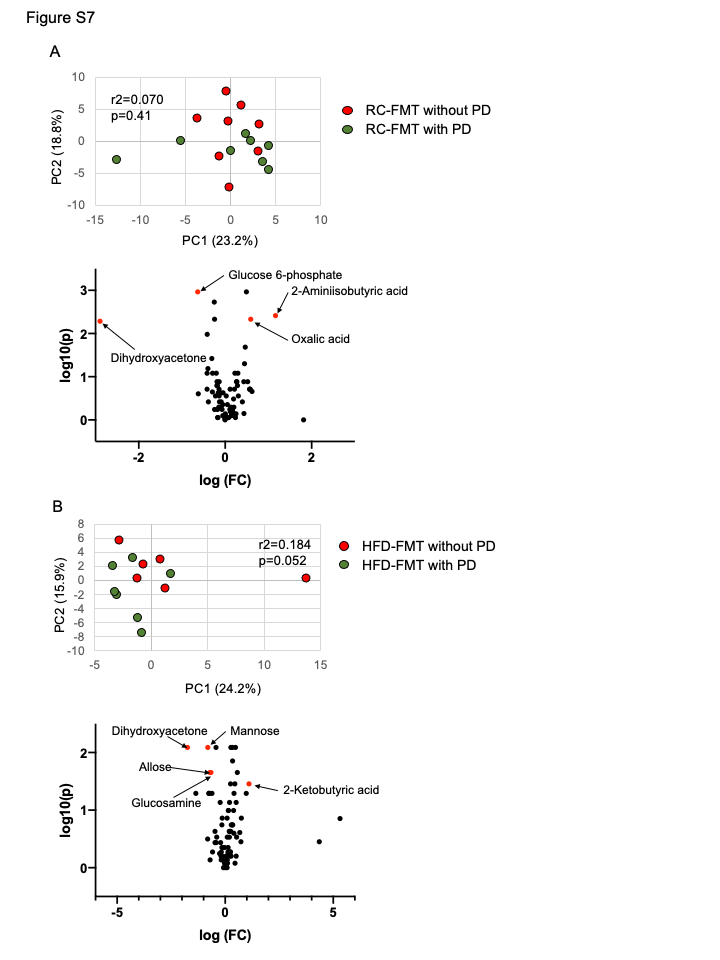

Supplement: FIG S7 [file mbio.00771-21-sf007.tif]

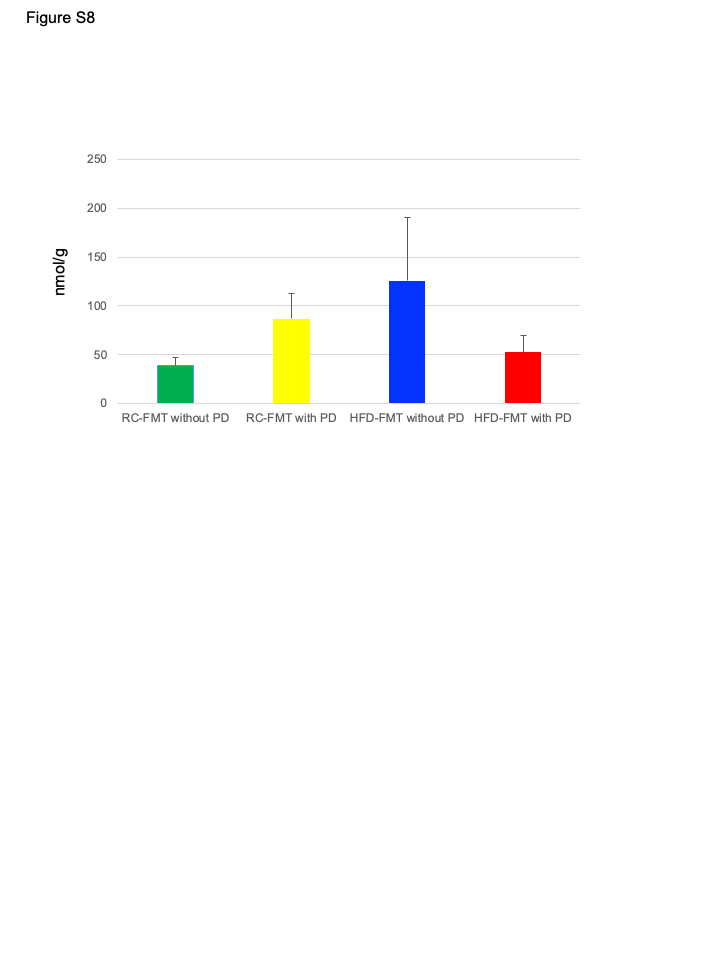

Supplement: FIG S8 [file mbio.00771-21-sf008.tif]
