# Supplementary material for: Obesity-Related Gut Microbiota Aggravates Alveolar Bone Destruction in Experimental Periodontitis through Elevation of Uric Acid
Source: mBio. 2021 Jun 1;12(3):e00771-21. doi: 10.1128/mBio.00771-21 (PMC8262938; doi:10.1128/mBio.00771-21)
Supplement: TABLE S1 [file mbio.00771-21-st001.docx]

Table S1

| Gene | Primer sequences | |
| --- | --- | --- |
|  | Forward | Reverse |
| *Gapdh* | TCAACAGCAACTCCCACTCTT | ACCCTGTTGCTGTAGCCGTAT |
| *Tnfa* | GATCGGTCCCCAAAGGGATG | TTGACGGCAGAGAGGAGGTT |
| *Il1b* | TGCCACCTTTTGACAGTGATG | AAGGTCCACGGGAAAGACCAC |
| *Il6* | CCAGAGATACAAAGAATGATGG | ACTCCAGAAGACCAGAGGAAAT |
| *Il17* | ATCCCTCAAAGCTCAGCGTGTC | GGGTCTTCATTGCGGTGGAGAG |
| *Il10* | CAGTGGAGCAGGTGAAGAGT | CAAGGAAGAACCCCTCCCATC |
